# Evaluation of a DNA-based method for spice/herb authentication, so you do not have to worry about what is in your curry, buon appetito!

**DOI:** 10.1371/journal.pone.0186283

**Published:** 2017-10-11

**Authors:** Maslin Osathanunkul, Sarawut Ounjai, Rossarin Osathanunkul, Panagiotis Madesis

**Affiliations:** 1 Department of Biology, Faculty of Science, Chiang Mai University, Chiang Mai, Thailand; 2 Center of Excellence in Bioresources for Agriculture, Industry and Medicine, Chiang Mai University, Chiang Mai, Thailand; 3 Faculty of Economics, Chiang Mai University, Chiang Mai, Thailand; 4 Institute of Applied Biosciences, Centre for Research & Technology Hellas (CERTH), Thessaloniki, Greece; Istituto di Biologia e Biotecnologia Agraria Consiglio Nazionale delle Ricerche, ITALY

## Abstract

It is long believed that some spices may help protect against certain chronic conditions. Spices are usually parts of plants that have been powdered into small pieces. Have you ever wondered what the curry powder in your dish is made of? The aim of this work was to develop an appropriate DNA-based method for assessment of spice identity. Selecting the best marker for species recognition in the Zingiberaceae family. Six DNA regions were investigated *in silico*, including ITS, *matK*, *rbcL*, *rpoC*, *trnH-psbA* and *trnL*. Then, only four regions (ITS, *matK*, *rbcL* and *trnH-psbA*) were included in the simulated HRM (High-resolution Melting) analysis as the results from previous analysis showed that *rpoC* and *trnL* may not be suitable to be used to identify Zingiberaceae species in HRM analysis based on both the percentage of nucleotide variation and GC content. Simulated HRM analysis was performed to test the feasibility of Bar-HRM. We found that ITS2 is the most effective region to be used for identification of the studied species and thus was used in laboratory HRM analysis. All seven tested Zingiberaceae plants were then able to be distinguished using the ITS2 primers in laboratory HRM. Most importantly the melting curves gained from fresh and dried tissue overlapped, which is a crucial outcome for the applicability of the analysis. The method could be used in an authentication test for dried products. In the authentication test, only one of seven store-sold Zingiberaceae products that were tested contained the species listed on their labels, while we found substitution/contamination of the tested purchased products in the rest.

## Introduction

Spices are primarily used for flavouring, colouring or preserving food, and they usually come from seeds, fruit, roots, bark, berries, and buds of plants. In addition to culinary use, spices are sometimes used in medicine, cosmetics or perfume productions and in religious rituals. Modern science has now shown that many of them do indeed carry remarkable health benefits. There has been great expansion of spicy products in the Thailand market as the number of products increased rapidly, particularly spices in the ginger or Zingiberaceae family, which includes several important spice producers. There are many plants in the Zingiberaceae family that have been deemed as economic crops that can generate national income, such as *Zingiber officinale*, *Curcuma longa*, *Curcuma zedoaria*, *Kaempferia parviflora*, *Boesenbergia rotunda*, *Zingiber montanum*, *Alpinia galangal* and *Amomum uliginosum* [1]. The external appearance of plants in the family is very similar, and identification based on morphological characters is sometimes difficult. Moreover, plants sold as spices are commonly powdered into small pieces, which also hampers morphological identification. Most spices are distributed in a processed form and it is very difficult to prove and identify their original species, but authentication of spice products is vital for both manufacturers and consumers.

Molecular identification through DNA barcoding is an efficient technique for the identification of plant species. A considerable amount of literature has actually been published, and it shows that DNA barcoding can be used to distinguish closely related medicinal plants [2–4]. The increasing and diverse applications of medicinal plant barcoding have been highlighted in several scientific reviews [5,6]. In addition, DNA barcoding is now recognized as a suitable molecular technology to greatly improve the traceability of food and drinks from producers to dining tables. Currently, this method continues to be largely exploited for genetic identification and adulteration detection in many plant species along with their food products [7]. However, DNA barcoding in plants does have limitations, including difficulty in DNA amplification due to degraded DNA in the processed samples [8], mismatches at binding sites such as *matK* [3,9,10], and low rates of discrimination capabilities, such as *rbcL* [4,11]. Developing and validating sequencing-free methods that are reliable and faster than DNA barcoding is challenging. Recent development of a DNA-based identification approach combines the advantages of two techniques, i.e., barcoding and high-resolution melting (HRM) analysis, called Bar-HRM [12–15]. This rapid growing sensitivity is likely to generate an increase in the speed and accuracy of species identification compared with morphological-based identification or using DNA barcoding alone.

Bar-HRM has been used in a number of comparable applications. Bar-HRM is revolutionising the way we can trace plant species in herbal, agricultural and food products; however, the approach faces challenges that can hinder our ability to produce robust results. Recently, a comprehensive evaluation of the use of Bar-HRM in plant species identification addressed the choice of markers used, which could lead to success in different plant groups [16]. Different primer sets in HRM analysis were found to be suitable for different plant groups. Here, we emphasize choosing the best marker for spice species in the Zingiberaceae family. The success of this study will highlight the potential of Bar-HRM as a rapid, sensitive, economical, high-throughput and taxonomical expertise-free technique for routine identification of spice identity as a quality assurance approach. This will lead to creating standards for raw material inspection and spice processing manufacturing standards for consumer confidence, as well as quality assurance of Thai spicy products for export. Spices can then be developed as significant economic crops to generate national income.

## Materials and methods

### DNA mining of barcode regions

**Fig 1:**
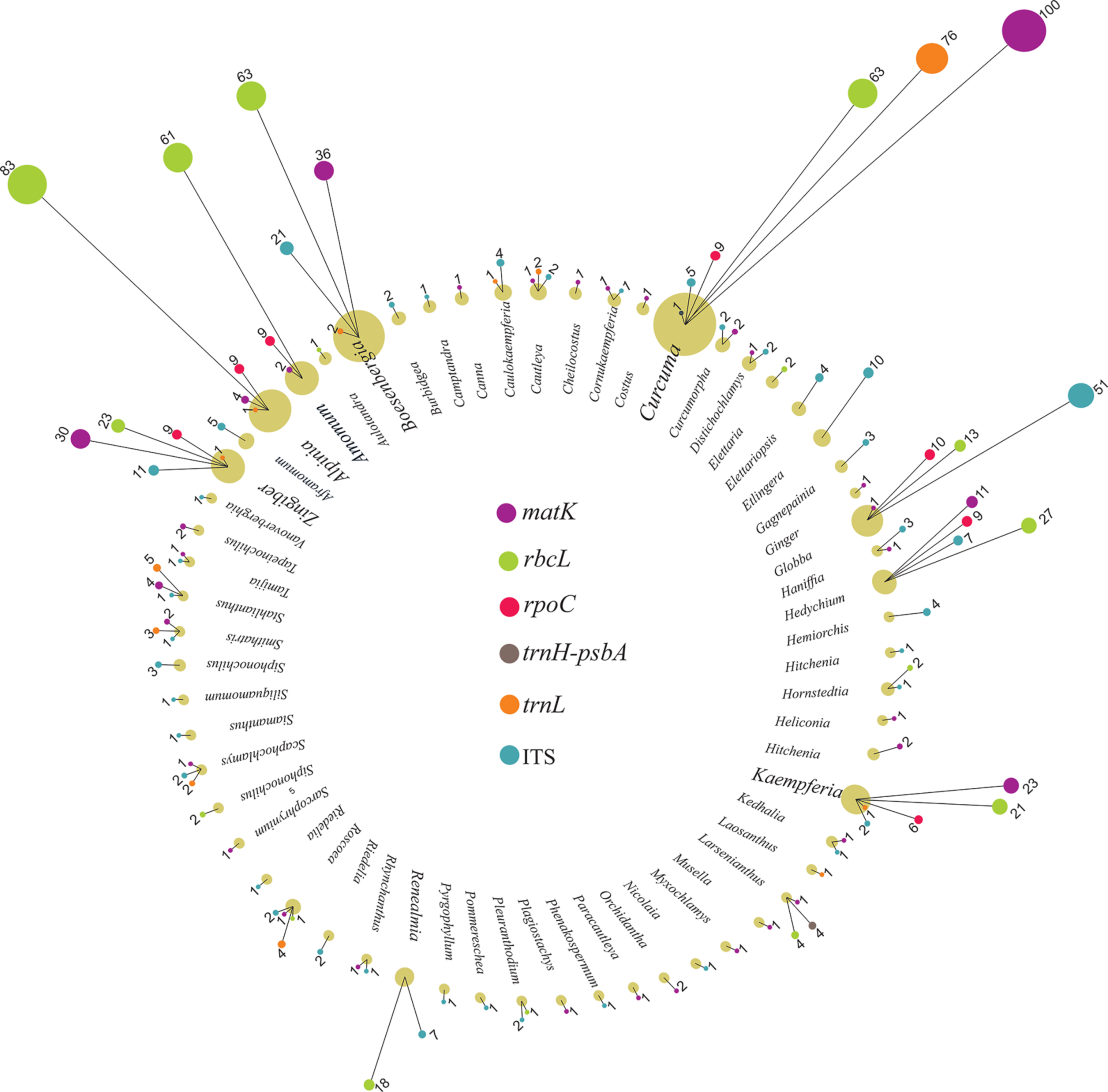
Retrieved sequences of Zingiberaceae from GenBank of six selected regions, including *matK*, *rbcL*, *rpoC*, *trnH-psbA*, *trnL* and ITS. Numbers of sequences from each genus are indicated.

To address the most suitable markers for identification of spices in Zingiberaceae based on the Bar-HRM technique, a few datasets were constructed to conduct a sequence profile analysis. The first dataset was used to evaluate the sequence profile of ITS, *matK*, *trnH*-*psbA* spacer region, *rbcL*, *rpoC* and *trnL* retrieved from GenBank, which included sequences from the entire family of Zingiberaceae (in total of 3,377 sequences from 62 genus) (Fig 1). With the second dataset, we deepened our scope to evaluate the sequence profile of amplicons generated by the Bar-HRM primers with only seven spice species in Zingiberaceae (Table 1).

**Table 1:** Plants and spice products included in this study.

No.	Scientific Name	HRM analysis		Commercial product
		Simulated	Laboratory	
		Accession Number*	Voucher Number	
1	*Alpinia galanga* (L.) Willd.	-	QBG 3640	P6.0-P6.1, P6.3-P6.4
2	*Amomum krervanh* Pierre ex Gagnep.	FJ972779, JF953193, FJ972790, GQ118657	-	-
3	*Amomum uliginosum* K.D. Koenig	-	QBG 36908	P7.0-P7.1
4	*Boesenbergia rotunda* (L.) Mansf.**	AF478726, KM983426, HM749001	QBG 30400	P4.0-P4.6
5	*Curcuma longa* L.	JQ409924, AB551931, KC441134, KC597940	QBG 78245	P2.0-P2.6
6	*Curcuma zedoaria* (Christm.) Roscoe	JQ409976, AB047743, KC441189, KC597942	-	-
7	*Kaempferia parviflora* wall. Ex Baker	KU159396, KU159407, KX559433, X559391	QBG 46181	P3.0-P3.6
8	*Zingiber montanum* (Koenig) Link ex Dietr.	DQ064585, KC598023, KC598029, KC597868	QBG 78341	P5.0-P5.6
9	*Zingiber officinale* Roscoe	KR816715, KC598025, GU180524, EU552521	QBG 46145	P1.0-P1.6
10	*Zingiber zerumbet* (L.) Roscoe ex Sm.	KC582875, HM367680, KC598032, KC597871	-	-

*Accession Number of sequences from ITS, *matK*, *rbcL*, and *trnH-psbA* regions, respectively

** *rbcL* sequence of the species was not included in the simulated HRM analysis due to lack of sequence in database (GenBank)

Sequences of ITS, *matK*, *trnH-psbA* spacer region, *rbcL*, *rpoC* and *trnL* were extracted from GenBank using the keyword ‘Zingiberaceae + (region title)’ at the end of September 2015. Some of the sequences retrieved from public databases, including GenBank, were found to be low quality with no known associated herbarium vouchers. For this reason, all of the sequences were subjected to critical evaluation and any low-quality sequences were removed. Criteria used to filter the sequences were as follows: (1) sequences were not ‘unverified’ without a species name, (2) sequences contained <3% ambiguous base ‘N’, and (3) a maximum of 3 samples (sequences) were included from a species. After processing, multiple alignments were made from the selected sequences using MEGA6 [17], and the sequence length (bp), conserved sites (%), variable sites (%), and GC content (%) of each data set were recorded.

### Plant material and DNA isolation

In laboratory analyses, seven Zingiberaceae spices with economic significance, including *Alpinia galangal*, *Amomum uliginosum*, *Boesenbergia rotunda*, *Curcuma longa*, *Kaempferia parviflora*, *Zingiber montanum* and *Zingiber officinale*, were investigated. Dried and fresh plant tissues for DNA extraction were kindly provided by the Queen Sirikit Botanic Garden (QSBG) and the faculty of pharmacy, Chiang Mai University, respectively (Table 1). The plant material was ground with liquid nitrogen, and 100 mg of fine powder was then used for DNA extraction with the Nucleospin Plant II kit (Macherey-Nagel, Germany) following the manufacturer’s instructions. The DNA concentration was estimated by standard spectrophotometric methods at 260 nm and 280 nm UV length by BioDrop (UK) and adjusted to a final concentration of 30 ng/μL. The DNA was stored at −20°C for further use.

### Simulated High Resolution Melting (HRM) analysis

Eight DNA sequences or fragments were selected based on their availability in all studied regions to test the feasibility of Bar-HRM. To determine the melting profile for each region, simulated HRM analyses were performed using melting curve predictions software called uMelt^SM^ [18] following the user guide (https://www.dna.utah.edu/umelt/docs/uMeltUserGuide.pdf). Species included in the assay were as follows: 1) *Amomum krervanh* 2) *Boesenbergia rotunda* 3) *Curcuma longa* 4) *Curcuma zedoaria* 5) *Kaempferia parviflora* 6) *Zingiber montanum* 7) *Zingiber montanum* and 8) *Zingiber zerumbet* (Table 1).

### Real-time PCR amplification and HRM analysis

To acquire the characteristic melting temperature (T_m_) that was capable of distinguishing the seven selected species of Zingiberaceae, PCR amplification using real-time PCR and DNA was performed using a Rotor-Gene Q 5plex HRM system (Qiagen, Australia) in a total volume of 20 μl on a plate. The reaction mixture for the real-time PCR and HRM analysis contained 10 μl of MeltDoctor HRM Master Mix (Applied Biosystems, USA), 1.2 μl of 10 mM forward primer, 1.2 μl of 10 mM reverse primer, 1 μl of 30 ng DNA and 6.6 μl of ddH_2_O. The primer pair was derived from the ITS2 sequence data retrieved from an online database (GenBank) (Forward 5’- CGCCTGCTTGGGCGTCATGGC -3’ and Reverse 5’- GGGCCTCGCCTGACTTGGGGCC -3’). Fluorescence dye was used to monitor both the accumulation of the amplified product and the high-resolution melting process in order to derive the T_m_ value during PCR.

The real-time PCR reaction conditions were as follows: an initial denaturing step at 95°C for 10 min, followed by 35 cycles of 95°C for 30 s, 57°C for 30 s, 72°C for 30 s, then a final extension step of 72°C for 5 min. The fluorescent data were acquired at the end of each extension step during PCR cycles. For the HRM experiments, fluorescence data were collected every 0.1°C. Rotor-Gene Q Series Software (version 2.3.1) was used to analyse the T_m_ and melting profile.

### Authentication of spice products

To further investigate the feasibility of Bar-HRM in species identification of Zingiberaceae spice products, the protocol was similar to the ‘Real-time PCR amplification and High-resolution Melting (HRM) analysis. Seven commercial products of each species, *Zingiber officinale* (P1.0-P1.6), *Curcuma longa* (P2.0-P2.6), *Kaempferia parviflora* (P3.0-P3.6), *Boesenbergia rotunda* (P4.0-P4.6), *Zingiber montanum* (P5.0-P5.6), and four of *Alpinia galangal* (P6.0-P6.1, P6.3-P6.4), two of *Amomum uliginosum* (P7.0-P7.1), were included in the analyses (Table 2).

**Table 2:** Store locations and products.

Store #	Store location (coordinates)	Product						
		*Z*. *officinale* (P1)	*C*. *longa* (P2)	*K*. *parviflora* (P3)	*B*. *rotunda* (P4)	*Z*. *montanum* (P5)	*A*. *galangal* (P6)	*A*. *uliginosum* (P7)
0	18.785149, 98.991650	**•**	**•**	**•**	**•**	**•**	**•**	**•**
1	18.791640, 98.998779	**•**	**•**	**•**	**•**	**•**	**•**	**•**
2	18.722076, 98.958554	**•**	**•**	**•**	**•**	**•**	x	x
3	18.787966, 98.991336	**•**	**•**	**•**	**•**	**•**	**•**	x
4	18.742528, 99.122746	**•**	**•**	**•**	**•**	**•**	**•**	x
5	18.735272, 99.017258	**•**	**•**	**•**	**•**	**•**	x	x
6	18.790268, 99.000587	**•**	**•**	**•**	**•**	**•**	x	x

● Products included in this study

X No products

## Results and discussion

### *In silico* analyses

The Zingiberaceae sequences from the selected barcode regions were extracted from GenBank, and the variable characters and average %GC content were calculated for all samples using MEGA6. HRM analysis is a method that is able to discriminate between DNA amplicons based on their, length, actual sequence and GC content. Sequence data were available for all selected markers. In total, 879 sequences of *matK*, 425 of *rbcL*, 60 of *rpoC*, 647 of *trnH-psbA* spacer region, 436 of *trnL* and 930 of ITS were retrieved, of which 242, 323, 60, 87, 102 and 252 sequences of each barcoding region were considered as being useful for further analysis (Table 3 and S1 Table). Most of the Zingiberaceae sequences found in GenBank belonged to genus *Curcuma* followed by *Alpinia*, *Amomum*, *Globba*, *Zingiber* and *Boesenbergia*, respectively (Fig 1). Five hundred and thirty-six variable sites (69.07%) were found inside the analysed ITS fragment (Table 4). The ITS sequences were found to be the most polymorphic, having the highest nucleotide differences, and the rank of the different DNA barcoding regions used in terms of nucleotide variation was found to be as follows: ITS > *matK* > *trnL* > *rbcL* > *trnH-psbA* > *rpoC*. The nucleotide variation within sequences is one of the key factors in HRM analysis as it influences the dissociation energy of the base pairs and result in different T_m_ values.

**Table 3:** Search results of the selected DNA regions of Zingiberaceae species retrieved from GenBank.

Regions	Number of		Length (bp)	
	Sequences	Genera	Min	Max
*matK*	879	48	209	3,315
*rbcL*	425	16	153	1,548
*rpoC*	60	7	501	501
*trnH-psbA*	647	4	153	1,243
*trnL*	436	35	191	1,197
ITS	930	50	208	2,661

**Table 4:** Zingiberaceae sequence profile of the selected regions (*matK*, *rbcL*, *rpoC*, *trnH-psbA*, *trnL* and ITS) in the analysis dataset.

Markers	*mat*K	*rbcL*	*rpoC*	*trnH-psbA*	*trn*L	ITS
Sequences in analysis dataset	242	323	60	87	102	252
Length (bp)	686	471	501	830	976	776
Variable sites (%)	36.88	12.95	3.79	11.69	16.49	69.07
Average %GC content	29.3	44.0	38.9	29.5	33.1	55.8

The average %GC content of all barcoding region amplicons was calculated, and thus, the predicted possible variation in the melting curves for the different markers was used. The *matK* region had the lowest average %GC content (29.3%), followed by *trnH-psbA*, *trnL*, *rpoC*, *rbcL* and ITS, with 29.5%, 33.1%, 38.9%, 44.0% and 55.8%, respectively. Based on the results above, it was estimated that the ITS would be a suitable barcoding marker for the HRM analyses for the target species. ITS has also been used for plant species identification with excellent results [2,5,19,20].

### Evaluation of markers for the discrimination of closely related Zingiberaceae species

**Fig 2:**
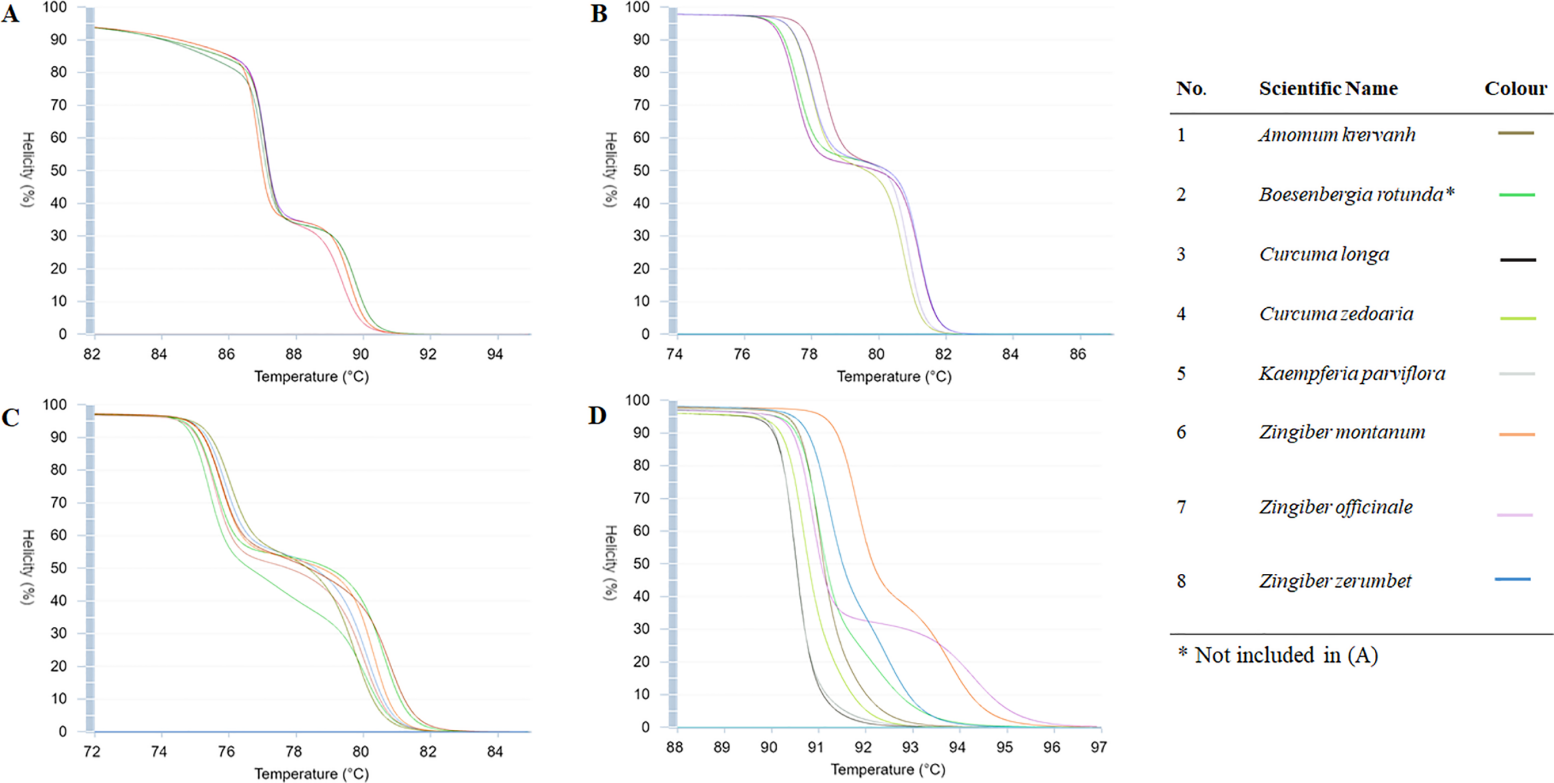
Simulated prediction of fluorescent high-resolution DNA melting curves of PCR products from (A) *rbcL* (B) *matK* (C) *trnH-psbA* and (D) ITS. Sequences or fragments of eight different plant species in Zingiberaceae were included in the analyses of three regions (*matK*, *trnH-psbA* and ITS), but only seven plant species of *rbcL* were included.

The DNA-fragments or sequences of selected Zinbearaceae species were analysed using a web-based application called uMelt^SM^ to define the discriminatory power via prediction of fluorescent high-resolution DNA melting curves of PCR products. Eight species (Table 1) were chosen for the analysis. Only four regions (ITS, *matK*, *rbcL* and *trnH-psbA*) were included in the simulated HRM analysis as the results from previous analysis showed that *rpoC* and *trnL* may not be suitable to be used to identify Zingiberaceae plants in HRM analysis based on both the percentage of nucleotide variation and the GC content. However, only seven sequences were used in the *rbcL* assay, as there is no sequence of *B*. *rotunda* that could be retrieved from the database (GenBank). Although we expected eight different melting curves from the eight tested species, as seen from Fig 2, none of the selected regions met our expectations. However, ITS and *trnH-psbA* showed the highest number of distinctive curves (seven), which made them the most promising regions for our study. Thus, we chose only ITS and *trnH-psbA* for further analyses.

### HRM analyses to identify Zingiberaceae species

#### Finding the most suitable marker

**Fig 3:**
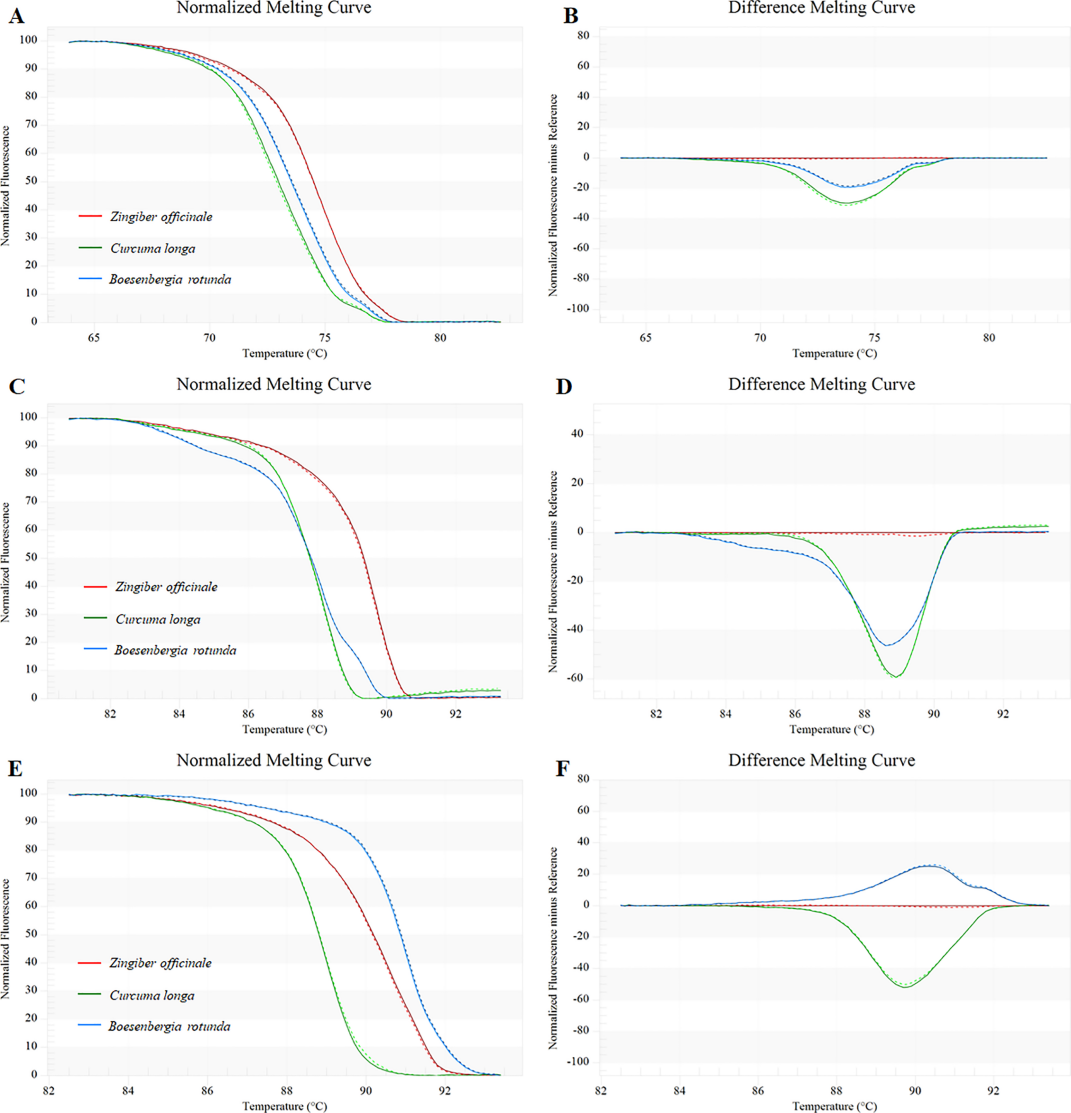
The melting profiles of amplicons generated from *trnH-psbA* (A-B), ITS1 (C-D) and ITS2 (E-F). The HRM analyses were performed in triplicate on each of the three taxa included *Zingiber officinale*, *Curcuma longa* and *Boesenbergia rotunda*. Fresh samples are represented with a thin line and the dried samples with a dotted line.

Three primer pairs were developed based on the retrieved sequences from GenBank, *trnH-psbA*, ITS1 and ITS2. To indicate the most suitable pair, HRM analysis was performed in triplicate on each of the three selected taxa (*Zingiber officinale*, *Curcuma longa* and *Boesenbergia rotunda*) to establish the T_m_ and melting profiles for each primer set. The DNA of each tested sample was isolated in triplicate and used in the HRM analysis. The melting profiles of all studied amplicons are illustrated in Fig 3A–3F. The mean of the melting temperatures obtained from each primer pair, along with the melting curve shape, was used to measure the discriminatory power of each region. Considering both T_m_ values and melting curves, all three primer pairs, *trnH-psbA* (Fig 3A and 3B), ITS1 (Fig 3C and 3D) and ITS2 (Fig 3E and 3F), could clearly differentiate the three tested plants species. The similar meting profiles of each tested species were obtained, thus indicating the feasibility of the method. Notably, both *trnH-psbA* and ITS are non-coding regions that normally contain high nucleotide variables. The *trnH-psbA* intergenic spacer is located in the chloroplast genome. This spacer has a high level of variation in the nucleotide sequences, and it was suggested as the barcode for plants by several research groups [21,22]. ITS, or internal transcribed spacer, is the non-coding region found in ribosomal DNA (18S-5.8S-26S rDNA) in the nuclear genome, which can be divided into ITS1 and ITS2. ITS1 is located between 18S rDNA and 5.8S rDNA, and ITS2 is located between 5.8S rDNA and 26S rDNA. Recently, ITS has been widely taken to be used in phylogenetic analysis. Moreover, a high level of nucleotide variation could be found in the ITS region, and it is also recommended for species identification in various plant groups [2,5,23].

Although the three tested primer pairs were efficient enough to differentiate the three selected species, the *trnH-psbA* region was reported to have high indels, and thus, there was inconstancy in the lengths of nucleotide sequences in the area of *trnH-psbA* in each group of plants (between 200 and over 1,000 base pairs) [22]. As amplicon length is one of major factors to be considered for HRM analysis, we did not choose *trnH-psbA* for the next analysis. When considering the differences in T_m_ values, melting profile and nucleotides variation (data not shown) of both ITS1 and ITS2, we only chose ITS2 for further experiments.

#### ITS2 and identification of seven popular Zingiberaceae spices

**Fig 4:**
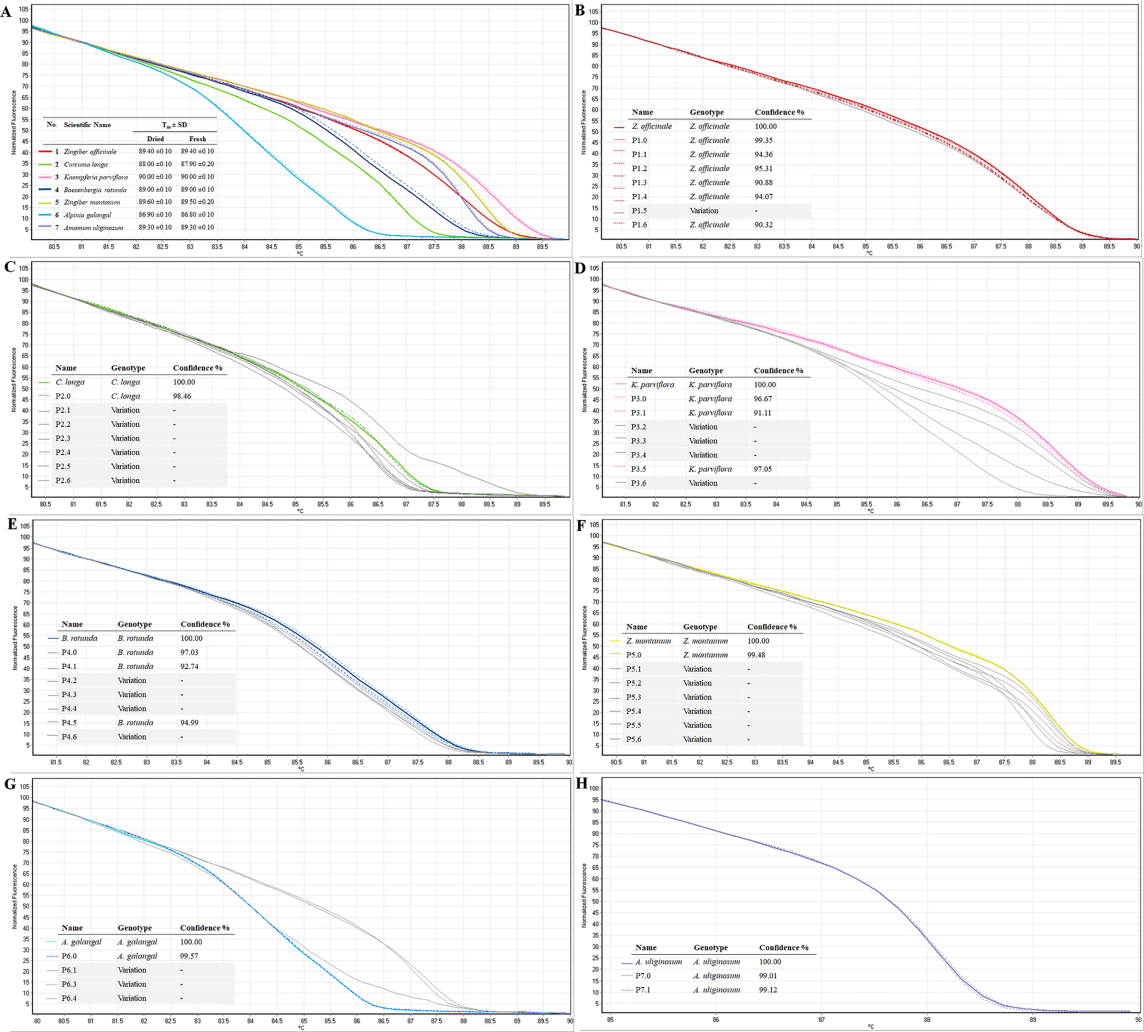
Melting profiles of ITS2 amplicons in HRM analyses, (A) of seven tested Zingiberaceae species. Fresh samples are represented with a thin line, and the dried samples with a dotted line. Average T_m_ values from three replicates of each plant species were presented. The fluorescent melting curves from ITS2 amplicons of spice products; (B) *Zingiber officinale*, (C) *Curcuma longa*, (D) *Kaempferia parviflora*, (E) *Boesenbergia rotunda*, (F) *Zingiber montanum*, (G) *Alpinia galangal*, and (H) *Amomum uliginosum* in an authentication assay. The coloured thin line indicates the reference sample of each plant species. The dotted lines with the colour as same as the reference samples indicate products containing the plant species as labelled. The grey lines indicate products that did not contain the plant species as labelled.

We used ITS2 primers in the HRM analysis in order to confirm its ability to differentiate the seven selected Zingiberaceae plants before using it to identify the species origin in spice products. The melting profiles of ITS2 amplicons from all tested species are shown in Fig 4A. The ITS2 primers produced unique fluorescence melting curves for each Zingiberaceae species. Similar melting curves were achieved from the same species regardless of whether the DNA template was extracted from fresh or dried tissues (Fig 4A). This is an important finding in view of the applicability of the analysis. Thus, there would be no problem in using the approach for identification of species in spice powders or to verify spice products sold in the markets.

The ITS2 primer pair was then used in HRM analysis for authentication to see whether they were consistent with the species indicated by the sellers. The tested spice products were purchased from various stores or producers in northern Thailand. HRM analysis was performed in triplicate on each of the tested products to establish the melting profiles. The shapes of the melting curves were analysed using a Rotor-Gene Q Series Software (version 2.3.1) to indicate plant species contained in the products. The melting profiles of all product amplicons are illustrated in Fig 4B–4H. HRM can detect differences among samples of as little as one base pair, however, in some cases, intra-specific and geographical variations within species exist in some plant groups. An arbitrary confidence interval cut-off of 90% was then chosen based on previous molecular studies. The results of the analyses shown in Table 5 reveal that more than half of the tested samples (24 of 41 products) may be contaminated or substituted with other species. Notably, only one of each *C*. *longa* and *Z*. *montanum* products (P2.0 and P5.0) presented similar melting curves to the reference species and thus implied that the products contained the indicated species. In addition, only one store (of seven) in this study, sold all tested spices that were identified as the same species indicated by the seller or on the labels (P1.0, P2.0, P3.0, P4.0, P5.0 P6.0 and P7.0).

**Table 5:** Bar-HRM identifications of the tested spice products.

Species on the label	Product code*	Bar-HRM	Species on the label	Product code	Bar-HRM
*Zingiber officinale*	P1.0	**•**	*Boesenbergia rotunda*	P4.0	**•**
	P1.1	**•**		P4.1	**•**
	P1.2	**•**		P4.2	X
	P1.3	**•**		P4.3	X
	P1.4	**•**		P4.4	X
	P1.5	X		P4.5	**•**
	P1.6	**•**		P4.6	X
*Curcuma longa*	P2.0	**•**	*Zingiber montanum*	P5.0	**•**
	P2.1	X		P5.1	X
	P2.2	X		P5.2	X
	P2.3	X		P5.3	X
	P2.4	X		P5.4	X
	P2.5	X		P5.5	X
	P2.6	X		P5.6	X
*Kaempferia parviflora*	P3.0	**•**	*Alpinia galangal*	P6.0	**•**
	P3.1	**•**		P6.1	X
	P3.2	X		P6.3	X
	P3.3	X		P6.4	X
	P3.4	X	*Amomum uliginosum*	P7.0	**•**
	P3.5	**•**		P7.1	**•**
	P3.6	x			

● Results from the Bar-HRM analysis indicated the same plant species as on the label

x Results from the Bar-HRM analysis indicated different plant species as labelled

* Products purchased from the same store were given the same number after ‘.’(dot) in product code

Spices were the main focus of our study as they were not only used for medicinal purposes but also serve as aromatherapy products, natural pest repellents, skin soothers, and even weapons (pepper spray). Several studies have shown that substitution of plant species occurs in herbal medicines. This could pose a serious problem in quality control in production, especially for those with high consumption and economic value. Only 41.46% of the tested spices here were found to be consistent with the species indicated by the sellers or labels. Whether intentional or not, substitution, contamination and adulteration of species should not happen. As most of the spices are sold in powdered form, it is hard to tell whether a product is actually the indicated species based on visual inspection. In addition, the Bar-HRM was previously reported to be able to discriminate an herbal mixture at various concentrations (1%–50%) [16]. Thus, the Bar-HRM has great potential for the identification of mixed spices.

## Conclusions

Several scientific research reports have noted that spices have health benefits, such as boosting the immune system, and they may help protect against certain chronic conditions, such as cancer, diabetes, and heart disease. Many traditional remedies have hinged on the use of spices as well, so they have always been in high demand. Before selling, most spices are powdered into small pieces, so it is difficult to identify the species by using morphological approaches. For detecting substitution and adulteration in spice products using molecular techniques, the Bar-HRM, has a number of advantages: (1) the HRM analysis method is highly sensitive for detecting a 1%–0.1% presence of an adulterated sample; (2) it is a high throughput technique that is capable of analysing multiple samples at the same time; and (3) no post-PCR processes are needed, and thus, cross-contamination can be avoided.

## Supporting information

S1 Table Zingiberaceae sequences of *mat*K, *psb*A-*trn*H, *rbc*L, *rpo*C, *trn*L and ITS were retrieved from GenBank (NCBI) for each genus with an accession number. (DOCX)
