# Supplementary material for: Evaluation of a DNA-based method for spice/herb authentication, so you do not have to worry about what is in your curry, buon appetito!
Source: PLoS One. 2017 Oct 11;12(10):e0186283. doi: 10.1371/journal.pone.0186283 (PMC5636142; doi:10.1371/journal.pone.0186283)
Supplement: S1 Table — (DOCX) [file pone.0186283.s001.docx]

**S1 Table.** Zingiberaceae sequences of *mat*K, *psb*A-*trn*H, *rbc*L, *rpo*C, *trn*L and ITS were retrieved from GenBank (NCBI) for each of the genus with accession number.

| **Genus** | **Accession number** | | | | | |
| --- | --- | --- | --- | --- | --- | --- |
|  | ***mat*K** | ***rbc*L** | ***rpo*C** | ***psb*A-*trn*H** | ***trn*L** | **ITS** |
| *Aframomum* | **-** | **-** | **-** | **-** | **-** | AF478704 - AF478708 |
| *Alpinia* | GU180389, KC597953,  KC597954, KC597956, | AY298816, GQ436403,  GQ436404, GQ436411,  GQ436412, GU180507,  GU180508, HM849760,  GU180511 - GU180514  GU180516, GU180517,  GU180538 - GU180541,  JF940725 - JF940740,  JF940744 - JF940750,  JF940752 - JF940761,  JF940763 - JF940765,  JF940768 - JF940772,  JF940774 - JF940776,  JF940778 - JF940781,  KC598059 - KC598066,  KF304124 - KF304132 | KF304244 - KF304252 | JN043817, JN043821,  JN043859, JN043865,  JN043866, JN043823,  JN043826, JN043835,  JN043843, JN043844,  JN043846, JN043849,  JN043856, JN043861,  JN043872, JN043815,  JN043818, JN043819,  JN043822, JN043824,  JN043825, JN043836,  JN043827 - JN043834,  JN043837, JN043841,  JN043842, JN043845,  JN043847, JN043848,  JN043851- JN043855,  JN043857, JN043858,  JN043860, JN043862,  JN043864, JN043873  JN043868 - JN043871,  JN043876 - JN043878 | AY769797 | AF478709 - F478720  GU097441, GU097443,  JF423298 |
| *Amomum* | JF953184, KC597975 | - | KF304253 - KF304261 | JN043925 - JN043928,  JN043933 - JN043935,  JN043949 - JN043957,  JN043963 - JN043965,  JN043967 - JN043971,  JN043976 - JN043978,  KJ151870 | - | AF414488, JF423302,  AF478721 - AF478724,  AY351985 - AY352012,  JF292430 |

**S1 Table. (cont.)** Zingiberaceae sequences of *mat*K, *psb*A-*trn*H, *rbc*L, *rpo*C, *trn*L and ITS were retrieved from GenBank (NCBI) for each of the genus with accession number.

| **Genus** | **Accession number** | | | | | |
| --- | --- | --- | --- | --- | --- | --- |
|  | ***mat*K** | ***rbc*L** | ***rpo*C** | ***psb*A-*trn*H** | ***trn*L** | **ITS** |
| *Aulotandra* | - | FN870769 | - | - | - | - |
| *Boesenbergia* | AF478826, AF478827,  JF715475 - JF715478,  JX992810 - JX992837,  JX992839, JX992840 | FJ972788 - FJ972793,  GU180509, GU180521,  JF940830 - JF940834,  JF940836 - JF940845,  JF940847 - JF940849,  JF940852 - JF940859,  JF940862 - JF940864,  JF940866 - JF940873,  KC597977, KC597978,  KF304133 - KF304141,  KJ151866 - KJ151869,  GU180522 | - | - | AY424778, AY424779 | AF478725 - AF478727,  JX992751 - JX992755, JX992792 - JX992798,  JX992804 - JX992808,  JX992748 |
| *Burbidgea* | - | - | - | - | - | AF478729, AF478728 |
| *Camptandra* | - | - | - | - | - | AF478730 |
| *Canna* | AF478906 | - | - | - | - | - |
| *Caulokaempferia* | - | - | - | - | AY424781 | AF478731 - AF478733 |
| *Cautleya* | AF478833 | - | - | - | AY424782, AY424792 | AF478735, AF478734 |
| *Cheilocostus* | JF715500 | - | - | - | - | - |
| *Cornukaempferia* | AF478835 | - | - | - | - | AF478736 |
| *Costus* | AF478907 | - | - | - | - | - |

**S1 Table. (cont.)** Zingiberaceae sequences of *mat*K, *psb*A-*trn*H, *rbc*L, *rpo*C, *trn*L and ITS were retrieved from GenBank (NCBI) for each of the genus with accession number.

| **Genus** | **Accession number** | | | | | |
| --- | --- | --- | --- | --- | --- | --- |
|  | ***mat*K** | ***rbc*L** | ***rpo*C** | ***psb*A-*trn*H** | ***trn*L** | **ITS** |
| *Curcuma* | AB047731 - AB047736,  AB047738 - AB047741,  AB047743 - AB047747,  AB047749 - AB047752,  AB551929 - AB551932,  AB649974, HM367646,  AF478836 - AF478841,  GQ248110, GQ434104,  GQ434106, GU180390,  GU180393 - GU180395,  GU180403, GU180404,  GU180410 - GU180412,  GU180416 - GU180419,  HM016510, HM016511,  JN180531 - JN180538,  JQ409655, JQ409660,  JQ409664 - JQ409666,  JQ409675 - JQ409678,  JQ409680 - JQ409693,  JQ409695, JQ409697,  JQ409700 - JQ409702,  JQ409704, JQ480152,  JQ409710 - JQ409713,  KF304022 - KF304030 | EF590515, EF590516,  GQ248582, GQ248583,  GQ436405, GQ436406,  GQ436413, GU180515,  GU180518, GU180519,  GU180528 - GU180531,  GU180535 - GU180537,  GU180542 - GU180545,  JF719548 - JF719564,  JF719569 - JF719575,  JF719577, JN180554,  JN180547 - JN180552,  KF304142 - KF304150,  KF432038 | KF304262 - KF304270 | KJ025042 | AY424785, DQ666419,  DQ471960 - DQ471978,  JQ409786 - JQ409788,  JQ409790 - JQ409796,  JQ409798, JQ409800,  JQ409802 - JQ409840,  JQ409847 - JQ409850 | AF478737, KJ461762,  AF478739 - AF478741 |
| *Curcumorpha* | AF478842, AF478843 | - | - | - | - | AF478743, AF478742 |
| *Distichochlamys* | AF478844 | - | - | - | - | AF478745, AF478744 |
| *Elettaria* | - | KC597986, KC597985 | - | - | - | - |
| *Elettariopsis* | - | - | - | - | - | AF478746 - AF478748,  AY352013 |
| *Etlingera* | - | - | - | - | - | AF414459, AF414460,  AF414474, AF414475,  AF414486, AF414505,  AF434895, AF478750,  AF478751, AY352014 |
| *Gagnepainia* | - | - | - | - | - | AF478752, AY339740 AY339704 |

**S1 Table. (cont.)** Zingiberaceae sequences of *mat*K, *psb*A-*trn*H, *rbc*L, *rpo*C, *trn*L and ITS were retrieved from GenBank (NCBI) for each of the genus with accession number.

| **Genus** | **Accession number** | | | | | |
| --- | --- | --- | --- | --- | --- | --- |
|  | ***mat*K** | ***rbc*L** | ***rpo*C** | ***psb*A-*trn*H** | ***trn*L** | **ITS** |
| *Ginger* | - | M91628 | - | - | - | - |
| *Globba* | AY341080 | AF243847, AF378777,  KF304151 - KF304159,  L05449 | KF304271 - KF304279 | - | - | AF478753 - AF478755,  AF478769, AF478770,  AY339665 - AY339703,  AY339707 - AY339727,  AY339729 - AY339739,  AY339741 - AY339743,  AF414500 |
| *Haniffia* | AF478855 | - | - | - | - | AF478756, JF825534 JF825533 |
| *Hedychium* | AB047754, AB049280,  AF478856 - AF478861,  GU180402, JN180506,  JN180510 | AF243848, GQ436407,  HM850043 - HM850045,  JN180604 - JN180609,  JN180611, JN180615,  JQ594501, L05450,  JX978632 - JX978634,  KF304160 - KF304168, | KF304280 - KF304288 | - | AY424787 | AF478757 - AF478762,  EF488010 |
| *Hemiorchis* | - | - | - | - | - | AF478764, AF478763 AY339706, AY339705 |
| *Hitchenia* | - | - | - | - | - | AF478765 |
| *Hornstedtia* | - | GU180548 | - | - | - | AF478766, AF414481 |
| *Heliconia* | AF478908 | - | - | - | - | - |
| *Hitchenia* | AF478864, AF478834 | - | - | - | - | - |
| *Kaempferia* | AB232053, AB232054,  AF478866, AF478867,  AF478869, GU180396,  GU180406 - GU180409,  KC597913 - KC597919,  KF304049 - KF304054 | GQ436409, GQ436410,  GU180532 - GU180534,  GU180546, GU180547,  KC597999 - KC598006,  KF304169 - KF304174,  GU180520, | KF304289 - KF304294 | - | AY424790 | AF478768, AF478767 |

**S1 Table. (cont.)** Zingiberaceae sequences of *mat*K, *psb*A-*trn*H, *rbc*L, *rpo*C, *trn*L and ITS were retrieved from GenBank (NCBI) for each of the genus with accession number.

| **Genus** | **Accession number** | | | | | |
| --- | --- | --- | --- | --- | --- | --- |
|  | ***mat*K** | ***rbc*L** | ***rpo*C** | ***psb*A-*trn*H** | ***trn*L** | **ITS** |
| *Kedhalia* | JF825540 | - | - | - | - | JF825535 |
| *Laosanthus* | - | - | - | - | JQ409845 | - |
| *Larsenianthus* | HM771405 | HM771401 - HM771403 | - | HM771396 - M771399 | - | - |
| *Musella* | AF478909 | - | - | - | - | - |
| *Myxochlamys* | AB553310 | - | - | - | - | - |
| *Nicolaia* | - | - | - | - | - | AF478749 |
| *Orchidantha* | AF478910, JF715499 | - | - | - | - | - |
| *Paracautleya* | HM367665 | - | - | - | - | - |
| *Paramomum* | - | - | - | - | - | AF478771 |
| *Phenakospermum* | AF478911 | - | - | - | - | - |
| *Plagiostachys* | - | GU180549 | - | - | - | AF478773, AF478772 |
| *Pleuranthodium* | - | - | - | - | - | AF478775, AF478774 |
| *Pommereschea* | - | - | - | - | - | AF478776 |
| *Pyrgophyllum* | - | - | - | - | - | AF478777 |
| *Renealmia* | - | HQ182449, JX978681,  JQ594504 - JQ594506,  JQ594508 - JQ594510,  JQ594577, JQ594578,  JQ594957, JX978680,  JX978683 - JX978688 | - | - | - | AF478778 - AF478783,  AF414476 |
| *Rhynchanthus* | AF478885 | - | - | - | - | AF478784 |
| *Riedelia* | - | L05460, AF243849 | - | - | - | - |
| *Roscoea* | AF478887 | JX903258 | - | - | AY424794 - AY424795  AY769781 - AY769782 | AF478787, AF478786 |

**S1 Table. (cont.)** Zingiberaceae sequences of *mat*K, *psb*A-*trn*H, *rbc*L, *rpo*C, *trn*L and ITS were retrieved from GenBank (NCBI) for each of the genus with accession number.

| **Genus** | **Accession number** | | | | | |
| --- | --- | --- | --- | --- | --- | --- |
|  | ***mat*K** | ***rbc*L** | ***rpo*C** | ***psb*A-*trn*H** | ***trn*L** | **ITS** |
| *Riedelia* | - | - | - | - | - | AF478785 |
| *Sarcophrynium* | AF478912 | - | - | - | - | - |
| *Siphonochilus* | - | FJ861127, AY656133 | - | - | - | - |
| *Scaphochlamys* | AF478890 | - | - | - | AY424796, AY424797 | AF478789, AF478788 |
| *Siamanthus* | - | - | - | - | - | AF478790 |
| *Siliquamomum* | - | - | - | - | - | AF478791 |
| *Siphonochilus* | - | - | - | - | - | AF478792 - AF478794 |
| *Smithatris* | AF478896, HM367645 | - | - | - | AY424798, JQ409797,  JQ409844 | AF478795 |
| *Stahlianthus* | AF478897, HM367672,  JF825538, JF825539 | - | - | - | AY424799, JQ409784,  JQ409785, JQ409842,  JQ409843 | AF478796 |
| *Tamijia* | JF715495 | - | - | - | - | AF478797 |
| *Tapeinochilus* | AF478913, JF715502 | - | - | - | - | - |
| *Vanoverberghia* | - | - | - | - | - | AF478798 |
| *Zingiber* | AF478900 - AF478903,  GQ434105, JF715496,  GU180398 - GU180401, GU180405, HM116892,  HM116900, HM116902,  HM116906, HM367673,  HM367674, JF715488,  KC598023 - KC598028,  KF304055 - KF304061 | AF243850, GQ436408,  GU180524 - GU180526,  JX978689, JX978690,  KC598029 - KC598034,  KF304175 - KF304183,  L05465 | KF304295 - KF304303 | - | AY424800 | - |
| *Zingiberaceae* | - | - | - | - | - | AF414499, KJ025068  AF478799 - AF478803,  JF825537, KC582864,  KC582866, KC582875 |
